# The *GmSNAP11* Contributes to Resistance to Soybean Cyst Nematode Race 4 in *Glycine max*

**DOI:** 10.3389/fpls.2022.939763

**Published:** 2022-07-04

**Authors:** Abdulwahab S. Shaibu, Shengrui Zhang, Junkui Ma, Yue Feng, Yuanyuan Huai, Jie Qi, Jing Li, Ahmed M. Abdelghany, Muhammad Azam, Honey Thet Paing Htway, Junming Sun, Bin Li

**Affiliations:** ^1^The National Engineering Research Center for Crop Molecular Breeding, MARA Key Laboratory of Soybean Biology (Beijing), Institute of Crop Sciences, Chinese Academy of Agricultural Sciences, Beijing, China; ^2^Department of Agronomy, Bayero University Kano, Kano, Nigeria; ^3^Institute of Industrial Crop Research, Shanxi Academy of Agricultural Sciences, Fenyang, China

**Keywords:** soybean cyst nematode resistance, bulked segregant analysis, candidate genes, diagnostic markers, GmSNAP, soybean (*Glycine max* L. Merrill)

## Abstract

Soybean cyst nematode (SCN) has devastating effects on soybean production, making it crucial to identify genes conferring SCN resistance. Here we employed next-generation sequencing-based bulked segregant analysis (BSA) to discover genomic regions, candidate genes, and diagnostic markers for resistance to SCN race 4 (SCN4) in soybean. Phenotypic analysis revealed highly significant differences among the reactions of 145 recombinant inbred lines (RILs) to SCN4. In combination with euclidean distance (ED) and Δsingle-nucleotide polymorphism (SNP)-index analyses, we identified a genomic region on Gm11 (designated as *rhg1-paralog*) associated with SCN4 resistance. Overexpression and RNA interference analyzes of the two candidate genes identified in this region (*GmPLAC8* and *GmSNAP11*) revealed that only *GmSNAP11* significantly contributes to SCN4 resistance. We developed a diagnostic marker for *GmSNAP11*. Using this marker, together with previously developed markers for SCN-resistant loci, *rhg1* and *Rhg4*, we evaluated the relationship between genotypes and SCN4 resistance in 145 RILs and 30 soybean accessions. The results showed that all the SCN4-resistant lines harbored all the three loci, however, some lines harboring the three loci were still susceptible to SCN4. This suggests that these three loci are necessary for the resistance to SCN4, but they alone cannot confer full resistance. The *GmSNAP11* and the diagnostic markers developed could be used in genomic-assisted breeding to develop soybean varieties with increased resistance to SCN4.

## Highlights

– We identified *rhg1-paralog* for the resistance to SCN4.
– *GmSNAP11* confers the major resistance of *rhg1-paralog* to SCN4.
– *rhg1*, *Rhg4*, and *rhg1-paralog* are necessary but cannot confer the full resistance to SCN4.

## Introduction

Soybean (*Glycine max* L. Merrill) is an important legume crop, providing approximately 68% of the world’s supply of protein-rich meals and serving as an excellent source of vegetable oil and renewable fuel. The average yield of soybean increased from 2,183 kg ha^–1^ in 1994 to 2,769 kg ha^–1^ in 2019 worldwide, with an annual increase of 23 kg ha^–1^ (FAOSTAT, 2020). However, biotic and abiotic constraints still limit soybean production (Hartman et al., 2011).

Soybean cyst nematode (SCN, *Heterodera glycines* Ichinohe) is an economically important pest for soybean causing estimated annual losses of billions of dollars worldwide (Koenning and Wrather, 2010). *H. glycines* is found in many countries, especially where soybeans are grown on a commercial scale, such as the United States and China (Shannon et al., 2004). The damage caused by SCN is particularly devastating because the aboveground symptoms are not always visible, but stunted roots and reduced nodulation decrease grain yield (Davis and Tylka, 2000; Niblack et al., 2006). Practices such as crop rotation and the use of SCN-resistant soybean varieties are the most effective strategies for SCN control (Davis and Tylka, 2000). In the main soybean production area of China, SCN races 1 (SCN1) and 3 (SCN3) are the predominant races in the Northeast Region while in the Huanghuaihai Region, SCN race 4 (SCN4) is the predominant race (Liu et al., 2019; Shaibu et al., 2020). SCN4 is the most virulent race, however, the genes underlying SCN4 resistance in soybean have not yet been fully identified (Liu et al., 2019).

Numerous SCN-resistant resources have been identified from the abundant soybean germplasm (Li et al., 1991; Arelli et al., 1997; Shaibu et al., 2020). Nevertheless, only a few of these resources have been used in soybean breeding because most of them carry undesirable traits that are difficult to improve using conventional breeding techniques (Li et al., 2014). The use of PI 88788 in SCN resistance breeding programs has led to SCN population shifts, resulting in the emergence of new SCN biotypes (Diers and Arelli, 1999; Vuong et al., 2010).

Soluble N-ethylmaleimide-sensitive factor attachment proteins (SNAPs) have been widely studied in plants and animals. SNAPs are involved in maintaining plasma membrane stability, vesicular trafficking, calcium-binding, cytokinesis, membrane repair, and human genetic diseases including certain cancers (Andrews and Chakrabarti, 2005; Schapire et al., 2008; El Kasmi et al., 2013; Südhof, 2013). In plants, an α-*SNAP* gene associated with disease resistance is present in most plant genomes (Lakhssassi et al., 2017). In soybean, various loci conferring resistance to SCN have been identified, including *rhg1* (Cook et al., 2012) and *Rhg4* (Liu et al., 2012). The *rhg1* locus contains *Glyma.18G022500*, which encodes an α-SNAP and is referred to as *GmSNAP18* (Liu et al., 2017). The *Rhg4* locus contains *Glyma.08G108900*, which encodes a serine hydroxymethyltransferase (SHMT) and is referred to as *GmSHMT08* (Liu et al., 2012). The *rhg1* is categorized into *rhg1-a* and *rhg1-b*; the *rhg1-a* acts additively with *Rhg4* to confer SCN resistance in Peking-type soybean lines, while *GmSNAP18* (*rhg1-b*) alone confers SCN resistance in PI 88788-type lines (Cook et al., 2012; Liu et al., 2012, 2017). However, both types of resistance require additional genes to confer a wider spectrum of resistance (Liu et al., 2019). For instance, the two major sources (Peking and PI 88788) of SCN resistance are susceptible to SCN4 (Niblack et al., 2006), the predominant race in HuangHuaiHai Region of China (Lian et al., 2017).

The *SNAP* gene family in soybean contains five members. *GmSNAP11* shares a strong similarity with *GmSNAP18* and is thought to additively contribute to SCN3 resistance (Li et al., 2016; Lakhssassi et al., 2017; Tian et al., 2019). However, at the time of conducting this research, further molecular evidence for the SCN4 resistance of *GmSNAP11* is lacking. Moreover, the resistance function of the *SNAP* family to SCN4 is rarely reported. Recently, a γ*-SNAP* gene family was also reported to contribute to SCN resistance (Butler et al., 2021). The resistance of soybean to SCN is highly complex, highlighting the need to identify additional sources of resistance in order to develop new resistant varieties (Liu et al., 2019). The development of such varieties is crucial for the long-term management of SCN (Shaibu et al., 2020).

Functional analysis of soybean genes requires methods for rapid mapping of the genes that control important agronomic traits. A few genes controlling various agronomic traits such as stem growth habit, seed number per pod, hard-seededness, and salt tolerance have been identified by positional cloning (Jeong et al., 2012; Guan et al., 2014; Ping et al., 2014; Sun et al., 2015). Traditional map-based cloning is time-consuming and usually low-throughput. Bulked segregant analysis (BSA) is a simple approach for quickly identifying loci governing traits of interest (Giovannoni et al., 1991; Michelmore et al., 1991). The BSA approach has been used to discover novel genomic regions or genes conferring disease resistance in several legumes (Singh et al., 2016; Pandey et al., 2017; Deokar et al., 2019; Luo et al., 2019).

In the present study, we performed BSA to discover novel genomic regions and identify candidate genes for SCN4 resistance in soybean. Two candidate genes in the *rhg1-paralog* locus (similar to *rhg1*), *Glyma.11g234400* (*GmPLAC8*) and *Glyma.11g234500* (*GmSNAP11*), were functionally verified by analyzing genetically transformed hairy roots. In addition to using previously reported kompetitive allele-specific PCR (KASP) markers for *rhg1* and *Rhg4*, we developed a novel diagnostic marker for *rhg1*-*paralog*. These markers could be used for the pyramiding of loci for SCN4 resistance in soybean via marker-assisted selection.

## Materials and Methods

### Plant Materials

*Glycine max cv.* Jindou 23 (JD23, accession number: ZDD23989) and Huipizhiheidou (HPD, accession number: ZDD02315), with susceptibility and resistance to SCN4, respectively, were crossed, and confirmed F_1_ plants were self-fertilized to F_10_ to develop a recombinant inbred line (RIL) population. The HPD landrace was used for gene cloning and vector construction for functional analysis of genes. Two RILs having *rhg1*^+^/*Rhg4*^+^ background were used for functional analysis of genes. RIL L99, which is susceptible to SCN4, was used for overexpression (OE) analysis; while L9, which is resistant to SCN4, was used for RNA interference (RNAi) analysis. 1025 Chinese soybean accessions were provided by the Research Group of Soybean Genetic Resources (Professor Lijuan Qiu’s lab), Institute of Crop Sciences, Chinese Academy of Agricultural Sciences. Of these accessions, PI 437654, PI 548402 (Peking), PI 90763, PI 88788, PI 209332, and PI 548316 (Cloud) are widely used as indicators to classify SCN HG type and parents to breed varieties with SCN resistance.

### Identification of Resistance to Soybean Cyst Nematode 4 (SCN4)

The reaction of the RILs and soybean accessions to SCN4 was determined using female index (FI) values at the Institute of Industrial Crop Research, Shanxi Academy of Agricultural Sciences, China. Mature female soybean cyst nematodes were harvested and the eggs were released as described previously (Arelli et al., 1997). The eggs were hatched at 26°C for a week. Infective Juvenile 2 (J2) nematodes were concentrated by centrifugation at 1000 rpm for 2 min to a final concentration of 1000 J2 mL^–1^. Each 2-mL aliquot of this inoculum was placed into a 2-cm hole beside the roots of a soybean seedling. Plant roots were individually washed with distilled water 38 days after inoculation. Mature females were counted under a stereomicroscope and the FI on each plant was calculated. Rating of resistance (FI < 10%) and susceptibility (FI > 10%) was used to classify the reactions as described by Schmitt and Shannon (1992) using the following equation:


$$F\,I=\frac{a\,v\,e\,r\,a\,g\,e\,n\,u\,m\,b\,e\,r\,o\,f\,f\,e\,m\,a\,l\,e\,s\,f\,r\,o\,m\,d\,i\,f\,f\,e\,r\,e\,n\,t\,i\,a\,l}{a\,v\,e\,r\,a\,g\,e\,n\,u\,m\,b\,e\,r\,o\,f\,f\,e\,m\,a\,l\,e\,s\,f\,r\,o\,m\,L\,e\,e}\times 100$$

### Construction of Sequencing Libraries and Sequencing

Genomic DNA was isolated from young leaves of the 145 RILs using a genomic DNAsecure plant kit (Tiangen, Beijing, China). The RILs were genotyped for *rhg1* and *Rhg4*. The previously reported KASP markers, GSM381 and GSM383, were used for *rhg1* detection (Shi et al., 2015). For *Rhg4* genotyping, we developed a modified KASP marker GSM150 based on a previously reported marker GSM191 (Shi et al., 2015), which has the same target SNP but different primer sequences with GSM191.

Based on the analysis of SCN4 resistance, some RILs in the *rhg1*^+^*Rhg4*^+^ background had different levels of SCN4 resistance. Two bulks were generated from RILs in the *rhg1*^+^*Rhg4*^+^ background, with 14 RILs each for susceptibility (S-bulk) and resistance (R-bulk) to SCN4. The RILs having FI values within the range of 1.1 to 7.5% were bulked together as R-bulk, while the RILs having FI values within the range of 50.3 to 105.7% were bulked together as S-bulk. Approximately 5 μg DNA samples from the two parental lines and the two bulks were used to construct paired-end sequencing libraries using the TruSeq Nano DNA HT Sample Preparation Kit (Illumina, CA, United States). The sequencing libraries were subjected to sequencing on an Illumina HiSeq 2500 platform to generate 150-bp paired-end reads.

To ensure the reliability of reads, the raw reads were processed through a series of quality control (QC) procedures using in-house C scripts to trim adaptors and remove low-quality reads based on the following criteria: (1) Removing reads with ≥ 10% unidentified nucleotides; (2) Removing reads with > 50% bases having phred quality score ≤ 10; (3) Removing reads with > 10 nucleotides aligned to the adapter, allowing ≤ 10% mismatches; (4) Removing putative PCR duplicates generated by PCR amplification in the library construction process. The clean reads were further rechecked for quality using FASTQC (V0.11.3). The Q30 (99.9% base call accuracy) and the GC content of the clean data were calculated. Consequently, high-quality reads were aligned and mapped to the *Glycine max* Wm82.a2.v1 reference genome from Phytozome^1^ using BWA (V0.7.10) with default parameters (Langmead and Salzberg, 2012). The percentage of mapped reads, the average depth, and the coverage ratio were calculated for each of parental lines and bulks. Genome Analysis Toolkit (GATK) HaplotypeCaller function was used to call SNPs and small indels across parental lines and bulks (McKenna et al., 2010). The SNPs were filtered using the GATK Variant Filtration protocol with the following settings: quality by depth (QD) < 4, mapping quality (MQ) < 40, depth of coverage (DP) < 4, MQRankSum < – 12.5.

### Identification of Genomic Regions and Candidate Genes for Soybean Cyst Nematode (SCN)

The two bulks were re-sequenced separately, and homozygous SNPs between the parental lines and high-quality SNPs were selected for SNP-index analysis. We used two methods to detect genomic regions associated with SCN resistance using 2,019,213 high-quality SNPs.


(1) E⁢u⁢c⁢l⁢i⁢d⁢e⁢a⁢n⁢D⁢i⁢s⁢t⁢a⁢n⁢c⁢e⁢(E⁢D)⁢E⁢D

$$=\sqrt{\begin{array}{c} {\left(A_{m\,u\,t}-A_{w\,t}\right)}^{2}+{\left(C_{m\,u\,t}-C_{w\,t}\right)}^{2}\\ +{\left(G_{m\,u\,t}-G_{w\,t}\right)}^{2}+{\left(T_{m\,u\,t}-T_{w\,t}\right)}^{2} \end{array}}$$

*Amut* is the frequency of the *A* base in the mutation pool, and *Awt* is the frequency of the *A* base in the wild pool; the same is true for *Cmut*, *Gmut*, and *Tmut*.

Single-nucleotide polymorphism (SNP) sites with different genotypes between the two pools were used to calculate the depth of each base in different pools, and the ED value of each locus was calculated as described by a previous study (Hill et al., 2013). The ED values were quintic powered to reduce the effects of noise and increase the effects of large ED values. As a complementary method to reduce the effects of noise, the LOESS fitted curves were calculated using the values of ED^5^ of all SNPs as described by Hill et al. (2013). The genomic median of all LOESS fitted values plus three standard deviations was set as a correlation threshold to select peak regions (Hill et al., 2013).


(2) Δ⁢(S⁢N⁢P-i⁢n⁢d⁢e⁢x)

Association analysis based on the SNP index is a method used to detect significant differences in genotype frequency between the pools, as indicated by ΔSNP-index, which is calculated as follows:


$$S\,N\,P\,i\,n\,d\,e\,x\,\left(a\,a\right)=\frac{M\,a\,a}{\left(M\,a\,a+P\,a\,a\right)}$$

$$S\,N\,P\,i\,n\,d\,e\,x\,\left(a\,b\right)=\frac{M\,a\,b}{\left(M\,a\,b+P\,a\,b\right)}$$

S⁢N⁢P⁢i⁢n⁢d⁢e⁢x=S⁢N⁢P⁢i⁢n⁢d⁢e⁢x⁢(a⁢a)-S⁢N⁢P⁢i⁢n⁢d⁢e⁢x⁢(a⁢b)

Maa represents the depth of aa from the maternal parent, Paa represents the depth of aa from the paternal parent, Mab represents the depth of ab from the maternal parent, and Pab represents the depth of ab from the paternal parent.

To reduce the number of false-positive loci, we employed a sliding-window method by calculating the average value of the SNP-index over a 4-Mb window, with an incrementing window of 10 kb (Fekih et al., 2013). The 99th percentiles of all fitted ΔSNP-index values were set as the threshold for identifying genomic regions.

Finally, the ED, SNP-index, and ΔSNP-index values were plotted, and the intersections between candidate regions that were identified using the ED and ΔSNP index methods were designated as final candidate SCN4 resistance-associated regions. We selected SNPs with non-synonymous, splice site acceptor and donor, start and stop codon, and gain and loss within these final candidate regions. Then we selected the SNPs from R-Bulk that was the same as that from HPD and the SNPs from S-Bulk that was the same as that from JD23. Furthermore, we analyzed the ΔSNP-index values of these SNPs and set the theoretical value of a causal SNP to be the third quartile value of their ΔSNP-index values. The regions for which ΔSNP-index values exceeded the threshold were considered candidate regions associated with resistance to SCN4. The identified genes were annotated against Nr, Swiss-Prot, and KOG/COG databases.

### Copy Number Analysis

Based on the high-quliaty clean reads, the read number for each base was used to represent its depth. The copy numbers of *rhg1* and *rhg1-paralog* were calculated using the average depth within the 31-kb region of *rhg1* and *rhg1-paralog*, as well as 31 kb upstream and downstream of *rhg1* and *rhg1-paralog*. Specifically, the ratios of the average depth of *rhg1* and *rhg1-paralog* regions to the average depth of their upstream and downstream regions were calculated separately. The copy numbers of *rhg1* and *rhg1-paralog* were estimated as the means of the two ratios compared to their upstream and downstream regions.

### Sequencing and Identification of Genetic Variation

The cDNA fragments of the two genes *GmPLAC8* (*Glyma.11g234400*) and *GmSNAP11* (*Glyma.11g234400*) from HPD were cloned and sequenced. Total RNA was isolated from young leaves and roots using TRIZOL kit (TransGen, Beijing, China), and cDNA was synthesized using a FastQuant RT kit (Tiangen, Beijing, China). RNA and cDNA quantities were measured using a NanoDrop spectrophotometer (Thermo Fischer Scientific, Langenselbold, Germany) and the quality of the RNA was checked by 1% agarose gel electrophoresis. PCR amplification was performed using KOD-Plus (Toyobo, Osaka, Japan) with the following cycling parameters: 35 cycles of 94°C 2 min (pre-denaturation), 94°C 15 s (denaturation), 55°C 30 s (annealing), and 68°C 2 min (extension) with 7 min of extension at 68°C. The PCR products were separated on a 1% agarose gel. The primers used for PCR are listed in Supplementary Table 1. The amplified fragments were purified using an EasyPure Quick Gel Extraction kit (TransGen, Osaka, Japan). The gel-purified fragments were cloned using a pEASY Blunt Cloning Kit (TransGen, Beijing, China) and cultured on a solid Luria-Bertani (LB) medium overnight at 37°C. PCR amplification was done to identify positive clones using a Taq kit (TransGen, Beijing, China). The PCR products were separated on a 1% agarose gel to identify positive clones with the correct amplicons. The positive clones were sequenced, and sequences that matched the original HPD sequence of *rhg1-paralog* were aligned to that from JD23.

The two genes in four SCN-resistant plant introduction lines were also cloned and sequenced, including lines PI 88788 (9 copies), PI 209332 (10 copies), PI 437654 (3 copies), and PI 548402 (3 copies). Total RNA was extracted from the samples using a TRIZOL kit (TransGen, Beijing, China), and cDNA was synthesized using a FastQuant RT kit (Tiangen, Beijing, China). The genes from the plant introduction lines were cloned and sequenced as described for HPD, and the sequences were aligned to HPD sequences to detect genetic variations.

### Protein Structure Modeling

The protein structure of GmSNAP11 for *cv.* HPD and JD23 were generated using SWISS-MODEL (Biozentrum^2^), and the resulting PDB files were analyzed with VectorNTI-3D Molecular Viewer software (Invitrogen Life Science Software, Frederick, MD).

### Functional Analyses of *GmPLAC8* and *GmSNAP11*

Functional analyses of the two SCN-resistant candidate genes identified in soybean (*GmPLAC8* and *GmSNAP11*) were performed using transgenic hairy roots. *GmSNAP18* was used as a control in all experiments. Plasmid DNA was purified from positive clones identified in overnight cultures of *Escherichia coli* in a LB liquid medium using a Tianprep Mini Plasmid Kit (Tiangen, Beijing, China). The pGFPGUS*plus* (13300 bp) vector (Vickers et al., 2007) was used for OE and RNAi studies. The pGFPGUS*plus* vector was firstly double digested with the restriction enzymes *Bgl*II and *Bst*EII. The coding sequences (CDS) of *GmPLAC8*, *GmSNAP11*, and *GmSNAP18* were then ligated into pGFPGUS*plus* to develop OE constructs using In-Fusion. Advantage PCR Cloning Kit (TAKARA, Tokyo, Japan). For the RNAi constructs, the RNAi structures for *GmPLAC8*, *GmSNAP11*, and *GmSNAP18* were separately ligated into pGFPGUS*plus*. The primers used to construct the RNAi and OE vectors are listed in Supplementary Table 1.

### Hairy Root Transformation

*Agrobacterium rhizogenes* strain K599 was co-bombarded with OE and RNAi constructs harboring the candidate genes using a Gene Pulser Electroporator (Bio-Rad Hercules, United States). The products were added to a yeast extract peptone (YEP) medium and shaken at 200 rpm at 28°C for one hour. Twenty μL of the product was inoculated onto a solid YEP medium and cultured overnight at 28°C. Positive clones were selected by PCR and stored in glycerol solution at -80°C for further analysis. The resistant (L9) and susceptible (L99) RILs were subjected to hairy root transformation as described previously (Chen et al., 2018). Transgenic hairy roots were identified by detecting green fluorescent protein with a LUYOR-3260 flashlight (LUYOR, Shanghai, China). Positive hairy roots were propagated twice and used for the SCN inoculation experiments.

### Nematode Infection of Transgenic Hairy Roots

Infective second-stage juveniles (J2) were used for this experiment. Nematodes were surface sterilized with sterilizing solution [0.004% (w/v) mercuric chloride, 0.004% (w/v) sodium azide, and 0.002% (v/v) Triton X-100] for 8 min followed by five washes with sterile water and resuspended in 0.1% (w/v) agarose. Hairy roots (3–4 cm) grown on ICM were inoculated approximately 1 cm above the root tip with 200 ± 25 J2s per root in a 25 mL volume. The plates were incubated in the dark for 30 days at room temperature. The experiment was conducted independently three times, and the number of cysts was counted under a stereomicroscope.

### RNA Isolation and qPCR

Total RNA was isolated from root tissues using a TRIZOL kit (TransGen, Beijing, China), and cDNA was synthesized using a FastQuant RT kit (Tiangen, Beijing, China). Quantitative RT-PCR was carried out using an Applied Biosystems QuantStudio 7 Flex Real-time PCR system (Applied Biosystems, CA, United States). Gene-specific primer pairs (Supplementary Table 1) were used, and all quantitative RT-PCR assays were carried out using three biological and three technical replicates. The soybean *Actin* gene was used as an internal control. PCR was performed using the following cycling parameters: 95°C for 3 min, 60°C for 30 s, 40 cycles of 95°C for 15 s, and 60°C for 1 min.

### SNP Marker Development and Validation of Candidate Genomic Regions

The KASP marker GSM151 for *rhg1-paralog* was designed based on the splice donor SNP in *GmSNAP11* (Supplementary Figure 1) and validated by Sanger sequencing of amplification products between *cv.* HPD and JD23. The KASP assay was also performed for *rhg1* and *Rhg4* loci to analyze the additive effects of these three loci. The KASP primers used in this study are shown in Supplementary Table 2. The KASP reaction mixture was prepared using the protocol described by LGC Genomics^3^. The following cycling conditions were used: 15 min at 95°C, followed by 10 touchdown cycles of 20 s at 94°C, 1 min at 65–57°C (dropping 0.8°C per cycle), 30 cycles of 20 s at 94°C, and 1 min at 57°C. The fluorescence levels were detected using a PHERAstar*^plus^* SNP Microplate Reader (BMG Labtech, Offenburg, Germany), imported using Kraken software by following the manufacturer’s guidelines, and analyzed using KLUSTERCALLER software (version 3.4.1.36; LGC Hoddesdon, United Kingdom). The KASP assay was firstly performed on 30 soybean accessions and 145 RILs, and then on 1025 Chinese soybean accessions. The 1025 soybean accessions and their origin were previously described in detail (Abdelghany et al., 2020). The geographical distribution of accessions with three *Rhg* loci (*rhg1*, *Rhg4*, and *rhg1*-*paralog*) in China was constructed with ArcGIS v10.0 (ESRI, Redlands, CA, United States).

## Results

### Phenotypic Variation and Construction of Extreme Bulks for Soybean Cyst Nematode 4 (SCN4) Resistance

The SCN4-resistance of a population of 145 recombinant inbred lines (RILs, F_2:10_) derived from a cross between soybean *cv.* Jindou23 (JD23, susceptible to SCN4) and Huipizhiheidou (HPD, resistant to SCN4) were evaluated. The distribution of the female index (FI) among the RIL populations was observed to follow a normal distribution (Supplementary Figure 2A), and highly significant differences (*P* < 0.0001) were observed in reactions to SCN4 among the RILs (Supplementary Figure 2B).

The genotypes of *rhg1* and *Rhg4* in the 145 RILs were determined using previously reported KASP markers, GSM381 and GSM383 (for *rhg1*), and a modified KASP marker GSM150 (for *Rhg4*), The RILs with the *rhg1^+^Rhg4*^+^ genotype exhibited various reactions to SCN4, ranging from resistant to susceptible (Supplementary Table 3), suggesting that additional alleles exist for this trait. Therefore, we selected 14 resistant and 14 susceptible lines with the *rhg1^+^Rhg4*^+^ genotype to prepare the resistant bulk (R-Bulk) and susceptible bulk (S-Bulk). The FI value ranged from 1.1 to 7.5% among the 14 resistant lines, and 50.3 to 105.7% among the 14 susceptible lines.

### Whole-Genome Resequencing, Reads Mapping, and Identification of SNPs

Whole-genome resequencing of the bulks was conducted and 183.37 Gb of reads were obtained. After filtering, 159.37 Gb of clean reads were obtained, including 43.91 Gb of clean reads for parental line JD23, 36.94 Gb for the S-Bulk, 46.87 Gb for parental line HPD, and 31.65 Gb for the R-Bulk with their Q30 values ≥ 88.44% (Supplementary Table 4). Over 98.6% of the clean reads could be mapped to the reference genome (w82.a2.v1). The average sequencing depth was 27 × and 32 × for the resistant and susceptible bulks, respectively, and 40 × and 41 × for parental line JD23 and HPD, respectively. More than 98% of the genome had at least 1 × coverage in all four samples (Supplementary Table 5). SNPs between the parental lines and the Williams 82 reference genome (W82.a2v1) were called and putative variations between the parental lines were identified by selecting SNPs that were unique to a single parent. Ultimately, 2,019,213 high-quality SNPs were identified between parental lines.

### Identification of Candidate Genomic Regions for Soybean Cyst Nematode 4 (SCN4) Resistance

**FIGURE 1:**
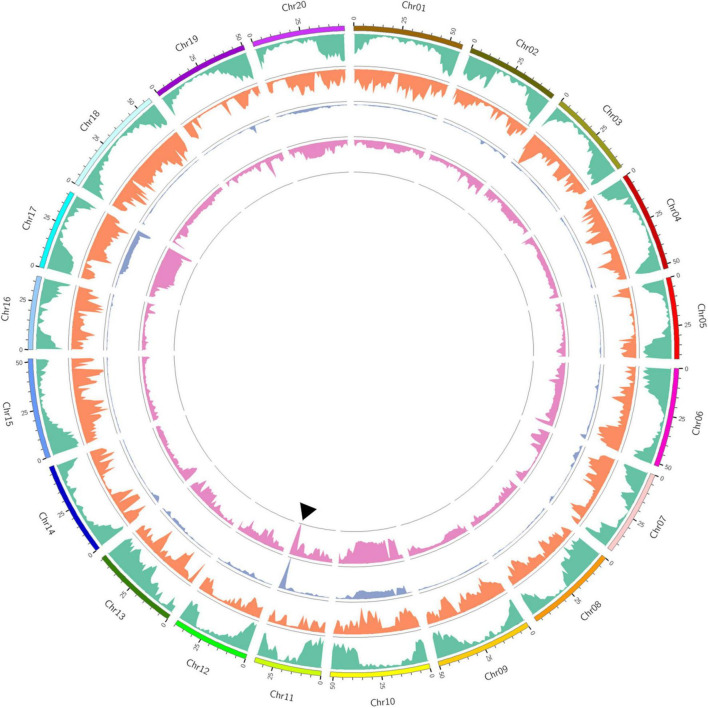
Bulked segregant analysis (BSA)-seq analysis of the recombinant inbred line (RIL) population. Outer to inner circles: chromosome distribution, gene distribution (green color), single-nucleotide polymorphism (SNP) density distribution (orange color), euclidean distance (ED) value distribution (cyan color), and ΔSNP-index distribution (rose color). The solid triangle indicates the peak position of the overlapping genomic regions at Gm11 using both ED and ΔSNP-index methods.

Euclidean distance (ED) analysis was performed using SNPs with different genotypes between the two bulks. Based on the threshold of 0.16, genomic regions on chromosomes 10, 11, and 17 were identified as important loci for SCN4 resistance, with a total genomic size of 24.31 Mb (Figure 1 and Supplementary Figure 3). A total of 2008 genes are included in these genomic regions (Supplementary Table 6).

Also, ΔSNP-index analysis was conducted to identify genomic regions associated with SCN4 resistance (Figure 1 and Supplementary Figure 4). According to the threshold value of 0.31, genomic regions on chromosome 07, 11, and 14 were identified for resistance to SCN4, with a total genomic size of 9.42 Mb. A total of 1452 genes were included in these genomic regions (Supplementary Table 7). By comparing the ED and ΔSNP-index results, an overlapping genomic region spanning 5.08 Mb on chromosome 11 was found to be associated with SCN4 resistance (Supplementary Table 8).

### Candidate Genes for Soybean Cyst Nematode 4 (SCN4) Resistance

Annotations of the genes identified in the genomic region on chromosome 11 were performed. In this region, we identified 8429 SNPs with different genotypes between the two parents. Most of these SNPs mapped to non-coding regions, such as intergenic regions, upstream and downstream regions, and introns of the annotated genes. However, we also identified 214 synonymous SNPs, 237 non-synonymous SNPs, 31 SNPs affecting splice sites, and 28 SNPs affecting start or stop codons in the coding regions of the genes (Supplementary Table 9). The non-synonymous SNPs and the SNPs affecting splice sites and start or stop codons were subjected to additional screening in which SNPs with different genotypes in the resistant parent and R-Bulk were filtered out. Ultimately, 46 non-synonymous SNPs, nine SNPs affecting splice sites, and seven SNPs affecting start codons were identified. We re-evaluated the ΔSNP-index values of these 62 SNPs and selected 15 SNPs with values higher than the third quartile value (0.692) due to their closeness to the theoretical value of a causal SNP (ΔSNP-index = 1).

The 15 SNPs were located in 12 annotated genes (Table 1). Surprisingly, of the 12 genes, two genes (*Glyma.11g234400* and *Glyma.11g234500*) had high similarity to the previously identified SCN resistance genes *Glyma.18G022600* and *Glyma.18G022500* at *rhg1*. *Glyma.11g234400* encodes (Z)-gamma-bisabolene synthase 1-related protein (a homolog of PLAC8 family protein) and *Glyma.11g234500* encodes an α-SNAP, therefore, we designated these two genes as *GmPLAC8* and *GmSNAP11*, respectively. The genomic region harboring *GmPLAC8* and *GmSNAP11* shows high collinearity with *rhg1*, the major SCN resistance locus; therefore, this region was designated as *rhg1-paralog*. We focused our analysis on the *rhg1-paralog* region and the two candidate genes.

**TABLE 1:** Annotation of the 12 candidate genes for resistance to soybean cyst nematode 4 (SCN4).

Gene ID	SNP location	JD23	HPD	ΔSNP-index	Annotation
*Glyma.11G221500*	31662968	C	G	0.85	Likely transcriptional regulator RABBIT EARS-like
*Glyma.11G221900*	31705035	G	A	0.82	Guanine-nucleotide exchange factor GNOM-like
*Glyma.11G233800*	32915089	C	A	0.71	Uncharacterized protein
*Glyma.11G234000*	32930334	T	G	0.75	Cation/H(+) antiporter 15-like isoform
	32932038	C	T	0.70	
*Glyma.11G234100*	32934646	T	C	0.74	Copper transport protein CCH-like isoform
*Glyma.11G234400*	32964982	G	A	0.78	PLAC8 family protein
	32965055	A	G	0.71	
*Glyma.11G234500*	32969916	C	G	0.79	Alpha-soluble NSF attachment protein 2-like
*Glyma.11G236100*	33099002	T	G	0.75	Gamma-glutamyltranspeptidase 1-like isoform
*Glyma.11G236300*	33115175	T	G	0.74	*Glycine max* bZIP transcription factor Bzip52
*Glyma.11G239000*	33338713	T	A	0.71	Putative ethylene insensitive 3-like 4 protein
*Glyma.11G239800*	33430048	C	T	0.79	ABC transporter D family member 1-like isoform
*Glyma.11G241700*	33584167	T	A	0.75	Histone H3.3-like
	33584159	C	T	0.71	

### Copy Number Variation in *rhg1-Paralog*

We examined the copy number of *rhg1-paralog* in various lines. No copy number variation within the 31-kb *rhg1-paralog* region was observed. There was only one copy of the 31-kb region of *rhg1*-*paralog* of JD23, HPD, R-Bulk, and S-Bulk (Supplementary Figure 5A), whereas there were three copies of *rhg1* on chromosome 18 in HPD, R-Bulk, and S-Bulk (Supplementary Figure 5B).

### Sequencing and Identification of *GmPLAC8* and *GmSNAP11* Genetic Variants

To identify the sequence variation between the resistant and susceptible parents, we isolated and cloned the cDNA sequences of the two newly identified genes from parental line HPD and aligned them with the sequences found in parental line JD23. For *GmPLAC8*, four SNPs and a 93-base pair (bp) deletion were detected in HPD (Supplementary Figure 6), while for *GmSNAP11*, three SNPs and a 17-bp insertion were detected in HPD (Supplementary Figure 7). Specifically, for *GmPLAC8*, the A/G SNP at position 1083 and the T/C SNP at position 1530 are synonymous, whereas the A/T SNP at position 886 and the G/A SNP at position 959 lead to Ala/Thr and Arg/Gln transitions at amino acid positions 296 and 320, respectively. The 93-bp deletion causes a 31-amino acid deletion (amino acids 368–398) in HPD. For *GmSNAP11*, the T/G SNP at position 108 and the G/A SNP at position 681 are synonymous, whereas the G/A SNP at position 535 causes an Ala/Thr transition at amino acid 179. The 17-bp insertion in HPD causes a frameshift mutation, generating a truncated GmSNAP11 protein (Supplementary Figure 8).

Alignment analysis between cDNA and genomic DNA of *GmSNAP11* from JD23 and HPD indicated that the insertion could be attributed to the above-mentioned SNP (Gm11: 32969916) discovered by BSA-sequencing. A G/T transition of this site causes an incorrect splice. As a result, a 17-bp fragment of the seventh intron in JD23 is retained in the *GmSNAP11* transcript for HPD, ultimately resulting in a frame-shift mutation. We cloned and sequenced these two genes in several resistant and susceptible varieties. For *GmSNAP11*, we detected similar sequences in four resistant plant introduction lines sequenced (Supplementary Figure 9). The 3D protein structure of GmSNAP11 showed significant variation between JD23 and HPD (Supplementary Figure 10).

### *GmSNAP11* Contributes to Soybean Cyst Nematode 4 (SCN4) Resistance

**FIGURE 2:**
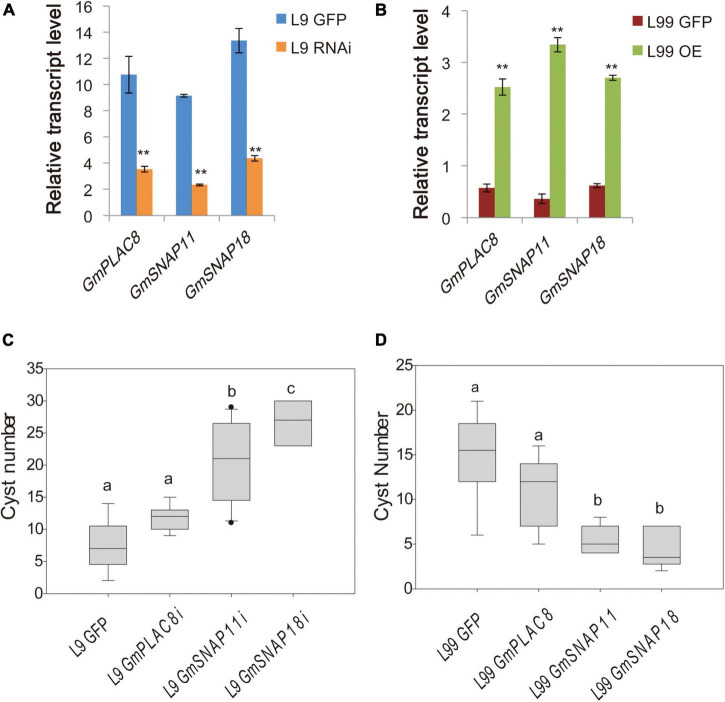
Relative transcript levels and cyst counts in various lines. **(A)** Relative transcript levels in the RNAi and **(B)** overexpression lines. The mean and SE of nine samples are shown. ** represents a significant difference at *P* < 0.01. **(C)** Cyst counts in soybean cyst nematode 4 (SCN4)-resistant RIL L9 (*rhg1*^+^, *Rhg4*^+^, *rhg1-paralog^+^*) with suppressed expression (RNAi) of these genes and **(D)** SCN4-susceptible RIL L99 (*rhg1*^+^, *Rhg4*^+^, *rhg1-paralog^–^*) overexpressing these genes. The different letters above the boxes represent different significant levels at *P* < 0.05.

To functionally validate the two candidate genes, we performed hairy root transformation of two RILs (L9 and L99) with the same *rhg1^+^Rhg4*^+^ backgrounds but different genotypes for *GmSNAP11* and *GmPLAC8* to alter the expression of the two genes (and *GmSNAP18* as a control). L9 (*rhg1*^+^, *Rhg4*^+^, and *rhg1-paralog^+^*) shows strong resistance to SCN4 (FI = 5.4), whereas L99 (*rhg1*^+^, *Rhg4*^+^, and *rhg1-paralog*^–^) was susceptible to SCN4 (FI = 66.5). Gene expression analysis indicated that the expressions of *GmPLAC8, GmSNAP11*, and *GmSNAP18* were partially suppressed by RNAi in transformed L9 hairy roots (Figure 2A). Highly significant differences (*P* < 0.0001) in the number of cysts were detected between L9 hairy roots transformed with different RNAi constructs (Figure 2C). The number of cysts was significantly higher when *GmSNAP11* expression was suppressed in the resistant line (Figure 2C). The highest number of cysts was observed in L9 hairy roots with suppressed *GmSNAP18* expression. By contrast, L9 hairy roots with partially suppressed *GmPLAC8* expression showed no significant difference in the average cyst number compared to the control (Figure 2C).

The overexpression of the candidate genes and *GmSNAP18* in the L99 (susceptible line) hairy roots showed that the expression levels are approximately 10-fold higher than that in the control (Figure 2B). The number of cysts significantly differed (*P* < 0.0001) among hairy roots transformed with different overexpression constructs (Figure 2D). Transgenic hairy roots overexpressing *GmSNAP18* had the fewest cysts (4.3), which was not significantly different from the number of cysts recorded in hairy roots overexpressing *GmSNAP11* (5.4) (Figure 2D). By contrast, the number of cysts in hairy roots overexpressing *GmPLAC8* was not significantly different (*P* > 0.05) from that of the control.

### The Additive Effects of Three Loci on Soybean Cyst Nematode 4 (SCN4) Resistance

To assess the additive effects of the three resistance loci (*rhg1*, *Rhg4*, and *rhg1-paralog*), we developed a diagnostic KASP marker for *GmSNAP11* (GSM151) that could successfully identify the SNP affecting the splice site (Supplementary Figure 11). This KASP marker clearly differentiated between the resistant and susceptible lines as well as their respective bulks. We tested this diagnostic marker, together with previously reported KASP markers for *rhg1* and *Rhg4*, by evaluating 30 soybean accessions and the 145 RILs for *rhg1* (*GmSNAP18*, Supplementary Figures 11A,B), *Rhg4* (*GmSHMT08*, Supplementary Figure 11C), and *rhg1-paralog* (*GmSNAP11*, Supplementary Figure 11D). All SCN4-resistant accessions harbored three resistance loci, while some accessions (e.g., Peking, PI 90763 and PI 84751) with three resistance loci were still susceptible to SCN4 (Table 2). Other accessions harborining one, two, or none of the resistant *Rhg* loci were susceptible to SCN4.

**TABLE 2:** Genotypes of three loci for soybean cyst nematode 4 (SCN4) resistance in 30 soybean accessions.

Accession	Genotype			SCN4 resistance
	
	*rhg1*	*Rhg4*	*rhg1-paralog*	
ZDD23181	R	R	R	R
ZDD10254	R	R	R	R
ZDD10251	R	R	R	R
ZDD02450	R	R	R	R
ZDD02258	R	R	R	R
ZDD02255	R	R	R	R
ZDD01861	R	R	R	R
ZDD03683	R	R	R	R
ZDD08494	R	R	R	R
ZDD02370	R	R	R	R
PI 437654	R	R	R	R
ZDD18394	R	R	R	S
ZDD08250	R	R	R	S
ZDD10293	R	R	R	S
ZDD01884	R	R	R	S
ZDD01890	R	R	R	S
ZDD08472	R	R	R	S
WDD03116	R	R	R	S
WDD00661	R	R	R	S
PI 84751	R	R	R	S
PI 548402 (Peking)	R	R	R	S
PI 90763	R	R	R	S
PI 88788	R	S	R	S
PI 209332	R	S	R	S
PI 548316 (Cloud)	R	S	R	S
ZDD23876	S	S	S	S
ZDD23989	S	S	S	S
ZDD24636	S	S	S	S
Hamilton	S	S	S	S
Lee	S	S	S	S

*R = resistant; S = susceptible.*

**FIGURE 3:**
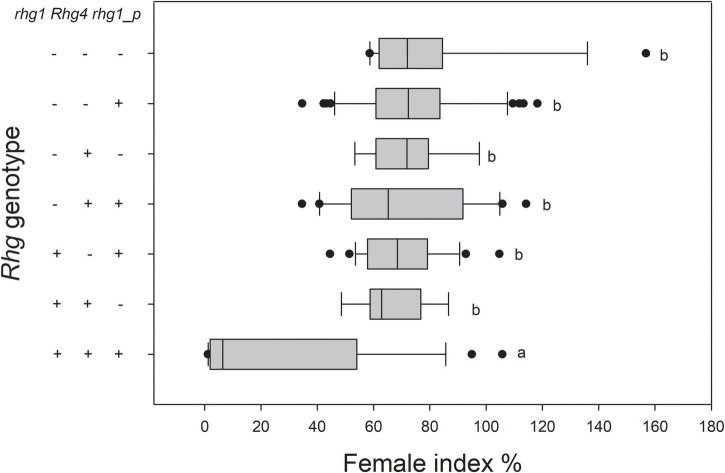
Reaction of RILs to SCN4 based on pyramiding of the *rhg1*, *Rhg4*, and *rhg1-paralog* loci (+ = present; - = absent). There were significant differences between the *Rhg* genotypes at *P* < 0.05. *rhg1-p* represents *rhg1-paralog.*

Among the 145 RILs, lines harboring *rhg1*, *Rhg4*, and *rhg1-paralog* showed significantly higher resistance to SCN4 than the other lines, although a wide range of resistance was observed among them. Whereas, no significant differences were observed among lines harboring one, two, or none of the resistance loci (Figure 3). Specifically, all 14 SCN4-resistant lines (FI ranging from 1.13–7.53) harbor *rhg1*, *Rhg4*, and *rhg1-paralog*, while 12 lines with all three genotypes were still susceptible to SCN4, with FI values ranging from 28.27–105.73 (Supplementary Table 3). Lines harboring only *rhg1* and *Rhg4* had FI ranging from 48.57–86.63, and lines harboring only *rhg1* and *rhg1-paralog* had FI ranging from 44.49–104.70. Lines with only the *rhg1*-*paralog* locus had FI ranging from 34.622–118.23. Lines lacking all three resistance loci had FI ranging from 58.6–156.77.

### Distributions of the Three *Rhg* Loci in China

**FIGURE 4:**
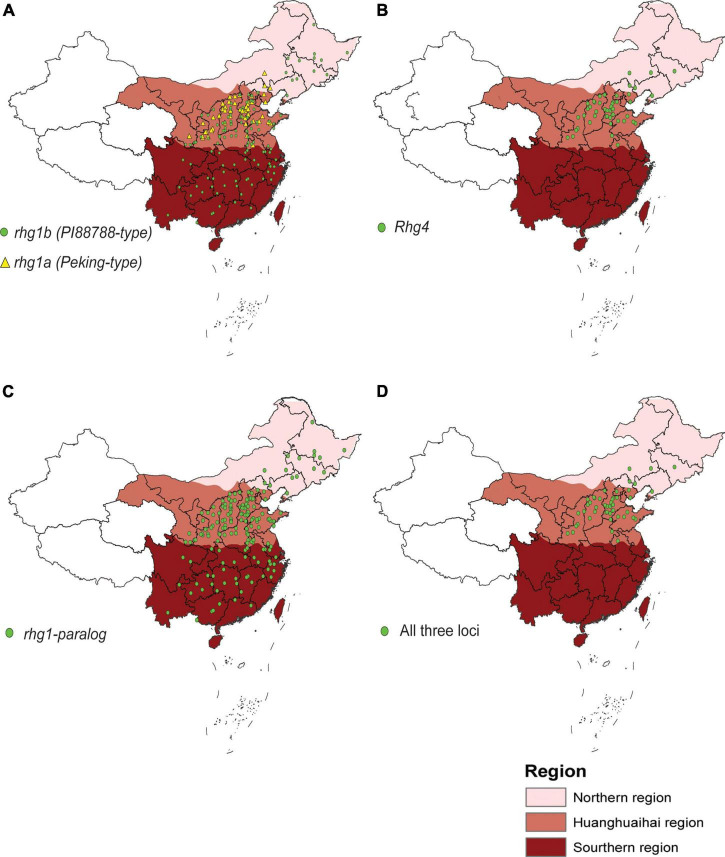
Map showing the distribution of resistance loci in 1025 Chinese accessions across Northern, Huanghuaihai and Southern regions of China. **(A)** *rhg1a* and *rhg1b*; **(B)** *Rhg4*; **(C)** *rhg1-paralog*; and **(D)** all three *Rhg* loci.

Finally, we examined the genotypes of the three resistance loci in 1025 Chinese accessions to explore their distribution in China. For *rhg1*, *rhg1b* is distributed across three major ecoregions in China, while *rhg1a* is primarily distributed across the Huanghuaihai Region of China (Figure 4A). The distribution of *Rhg4* is similar to that of *rhg1a* (Figure 4B), whereas *rhg1-paralog* shows a wide distribution across China (Figure 4C). Since SCN4 resistance at least relies on *rhg1*, *Rhg4*, and *rhg1-paralog*, we also mapped the distribution of the genotypes of accessions with all three resistance loci. As shown in Figure 4D, accessions with all three resistance loci are primarily distributed across the Huanghuaihai Region of China, where SCN4 is the predominant race of SCN. Additionally, these accessions with three loci were also evaluated for SCN4 resistance. As expected, 61% of these accessions exhibited resistance to SCN4, while 39% showed susceptible phenotype (Supplementary Table 10).

## Discussion

The damage caused by SCN is devastating and an established SCN population is difficult to eradicate. The two *Rhg* loci (*rhg1* and *Rhg4*) for SCN act additively to confer resistance, but this resistance is not sufficient. Another minor gene, *GmSNAP11*, has been assumed to have an additive effect with *GmSNAP18* in mediating SCN3 resistance (Li et al., 2016; Lakhssassi et al., 2017). However, SCN4 is the most virulent race, causing significant yield losses annually. The genes conferring resistance to SCN4 in soybean have not yet been fully identified or mapped (Liu et al., 2019).

Over the past few decades, map-based cloning is the most common approach for identifying and isolating candidate genes in many crops. The effectiveness of conventional mapping is limited because it is time-consuming, labor-intensive, and costly to develop marker sets (Lindner et al., 2012). Few genes have been functionally analyzed in soybean compared to other model species (Xia et al., 2013). In the present study, we successfully employed next-generation sequencing-based BSA to identify new genomic regions governing SCN4 resistance. Using this approach, we quickly associated loci with candidate genomic regions, greatly reducing the workload and saving time. We were also able to detect many SNPs and small indels between the parental lines and bulks. Indeed, causal mutations of candidate genes can be identified by detailed analysis of SNPs and indels in candidate genomic regions after fine mapping.

Resequencing of the R- and S-bulks revealed a causal region on chromosome 11 with a physical size of 5.08 Mb, which is very large, even with an average genome coverage of > 35 × for the two parental lines and bulks. However, similar coverage has been reported in soybean (Campbell et al., 2016; Dobbels et al., 2017; Song et al., 2017). Copy number variation is known to contribute to *rhg1*-mediated resistance to SCN (Cook et al., 2012). In the current study, copy number analysis showed that there was no copy number variation, but a splice donor SNP was identified for *GmSNAP11*. Therefore, we speculate that the SNP of *rhg1*-*paralog* rather than copy number variation contributes to SCN4 resistance. The structural variation in the 3D protein structure of GmSNAP11 between JD23 and HPD points to a clear difference in this protein between resistant and susceptible lines. Indeed, several studies have detected SNPs for *GmSNAP11* and suggested that this gene might function in SCN3 resistance by contributing additively to resistance with *GmSNAP18* (Li et al., 2016; Lakhssassi et al., 2017; Tian et al., 2019).

From the validation experiment using hairy root transformation, *GmPLAC8* did not appear to contribute to SCN4 resistance. Similar results have been previously reported for SCN3 resistance (Cook et al., 2014; Li et al., 2016). For *GmSNAP11*, however, the number of cysts in hairy roots increased significantly when the expression of this gene was suppressed. Also, overexpressing resistance-type *GmSNAP11* in susceptible lines significantly reduced the number of cysts compared to the control. *GmSNAP11* may function additively with *GmSNAP18* to mediate SCN3 resistance, as determined in previous studies (Lakhssassi et al., 2017; Tian et al., 2019). Specifically, the presence of the *GmSNAP11* resistance allele combined with *GmSNAP18* decreased the FI from 12.59% in lines having only *GmSNAP18* to 4.48% for SCN3 (Lakhssassi et al., 2017). Our study suggests *GmSNAP11* also confers the resistance to SCN4 in the presence of *rhg1* and *Rhg4*. Moreover, no significant difference between *GmSNAP18* and *GmSNAP11* cyst counts was observed for overexpressed transgenic lines. Both of them exhibited increased resistance to SCN4 when overexpressed constitutively even in the presence of *rhg1*, suggesting the abundance of resistant SNAPs (SNAP18 and SNAP11) may play the most important role for the resistance to SCN4. Since *GmSNAP11* presents a high sequence similarity with *GmSNAP18*, the CRISPR/Cas9-based gene editing will be employed in our subsequent study to exactly distinguish the effects between SNAP11 and SNAP18 on the resistance to SCN4.

It has been reported that the *GmSNAP18* and *GmSNAP11* are more highly related to each other than the other *GmSNAP* family members. Both genes usually cluster together in phylogenetic analysis, suggesting they are closely related (Lakhssassi et al., 2017). However, *GmSNAP18* is more closely related to the ancestral *SNAP* genes than *GmSNAP11*. Among *GmSNAP* genes, *GmSNAP11* and *GmSNAP18* are highly expressed in most tissues (Lakhssassi et al., 2017).

The α-SNAPs encoded by *rhg1* are unusual α-SNAP proteins that bind less well to wild-type N-ethylmaleimide-sensitive factor and can disrupt vesicle trafficking, eventually leading to cell death (Bayless et al., 2016). As the host syncytium develops, the relative abundance of *rhg1*-encoded defective α-SNAP variants strongly increases, which disrupts syncytium viability and restricts nematode growth and reproduction (Bayless et al., 2016). Furthermore, the α-SNAPs encoded by *GmSNAP18* and *GmSNAP11* are major sources of total α-SNAP proteins in soybean (Bayless et al., 2018).

Taking our observations together, considering the similar features of *GmSNAP11* and *GmSNAP18*, we propose that the mechanism by which *GmSNAP11* confers resistance to SCN is similar to that employed by *GmSNAP18*. We demonstrated that overexpressing *GmSNAP* (*GmSNAP11* and *GmSNAP18*) in the susceptible RIL (*rhg1*^+^*Rhg4*^+^*rhg1-paralog*^–^) reduced the cyst number (elevated resistance) when inoculated with SCN4. Therefore, we suggest that, like *GmSNAP18*, *GmSNAP11* confers resistance via the accumulation of impaired α-SNAP. Under SCN infestation, *GmSNAP18* and *GmSNAP11* are more highly expressed in most tissues and *GmSNAP18* is the most highly upregulated gene, followed by *GmSNAP11* (Lakhssassi et al., 2017). It is therefore expected that cultivars harboring both *GmSNAP18* and *GmSNAP11* would exhibit elevated resistance because their additive effect would lead to increased accumulation of impaired α-SNAP.

In the present study, we successfully transformed a SNP into a KASP marker and used it, along with two previously developed KASP markers, to examine the additive effects of *GmSNAP11*, *GmSNAP18*, and *GmSHMT08* on SCN4 resistance. The pyramiding effect of these genes on SCN4 seems more complicated than that on SCN3. Tian et al. (2019) showed that pyramiding these three loci conferred more resistance to SCN3 compared to lines containing only *GmSNAP18* and *GmSHMT08*. However, in this study, all SCN4-resistant RILs harbored all three genes, whereas several lines harboring the three genes were still susceptible to SCN4, suggesting that these three genes are necessary for the resistance to SCN4 but these genes alone cannot confer full resistance. Indeed, several studies have demonstrated that SCN resistance in soybean is a very complicated trait (Bayless et al., 2016, 2018; Lakhssassi et al., 2017; Liu et al., 2019; Patil et al., 2019). Several other genes might also contribute to the resistance to SCN3 (Tran et al., 2019) and SCN4 (Liu et al., 2019). The presence of other genes contributing to SCN resistance might explain why we observed variations in cyst number among the soybean accessions and RILs despite having similar pyramiding of the three *Rhg* loci. Nonetheless, the *rhg1*, *Rhg4*, and *rhg1*-*paralog* loci are major contributors to SCN resistance and our analysis highlighted the importance of *GmSNAP11* in contributing additively to SCN4 resistance. In addition, we demonstrated the potential of using these KASP markers to mine resistance alleles for SCN4 in breeding programs.

Analysis of the distribution of accessions with all three resistance loci suggested that the resistant genotypes originated from the Huanghuaihai Region of China. In the soybean production area of this region, SCN4 is the predominant race of SCN, but the genes conferring specific resistance to SCN4 have not yet been identified (Liu et al., 2019). The Huanghuaihai Region is seriously affected by SCN4, likely explaining why genotypes harboring the three major *Rhg* resistance loci evolved in this region.

## Conclusion

We successfully used next-generation sequencing-based BSA to identify novel genomic regions on chromosome 11 (designated as *rhg1-paralog*) for SCN4 resistance in soybean. Of the two genes identified, *GmSNAP11* was successfully validated as a gene that contributes additively to SCN4 resistance. *GmSNAP18* alone cannot confer sufficient resistance to SCN4, as additional genes acting singly or in combination with *GmSNAP18* are needed to enhance SCN4 resistance in soybean. *GmSNAP11* confers resistance to SCN4 via the accumulation of impaired SNAP, which is similar to the mode-of-action of *GmSNAP18*. Therefore, the α*-SNAP* gene family confers SCN resistance through the accumulation of impaired α*-*SNAP. The diagnostic marker developed for *GmSNAP11* in the current study and the existing markers for *rhg1* and *Rhg4* could be used for marker-assisted selection to identify germplasm with resistance at these loci and to enhance future breeding programs aimed at developing soybean varieties with strong resistance to SCN4.

## Data Availability Statement

The original contributions presented in the study are publicly available. This data can be found here: https://ngdc.cncb.ac.cn/, GVM000330.

## Author Contributions

BL and JS conceived, designed, and supervised the experiments. JM developed the RIL population and conducted field phenotyping. BL performed the BSA experiment. AS performed gene cloning, functional validation of the identified genes, performed the KASP experiment, interpreted the results, and prepared the draft manuscript. YF and YH assisted in vector construction and hairy root transformation. YF, AA, and MA extracted DNA from the accessions used for the KASP experiment. HH assisted with SCN inoculation and the KASP experiment. BL, JS, and SZ contributed to the improvement and final editing of the manuscript. All authors read and approved the final manuscript.

## Conflict of Interest

The authors declare that the research was conducted in the absence of any commercial or financial relationships that could be construed as a potential conflict of interest.

## Publisher’s Note

All claims expressed in this article are solely those of the authors and do not necessarily represent those of their affiliated organizations, or those of the publisher, the editors and the reviewers. Any product that may be evaluated in this article, or claim that may be made by its manufacturer, is not guaranteed or endorsed by the publisher.

## Footnotes

1
https://phytozome.jgi.doe.gov/pz/portal.html

2
https://swissmodel.expasy.org/

3
www.lgcgroup.com/genomics
