# Supplementary material for: The GmSNAP11 Contributes to Resistance to Soybean Cyst Nematode Race 4 in Glycine max
Source: Front Plant Sci. 2022 Jul 4;13:939763. doi: 10.3389/fpls.2022.939763 (PMC9289622; doi:10.3389/fpls.2022.939763)
Supplement: Supplementary file 1 [file Data_Sheet_1.docx]

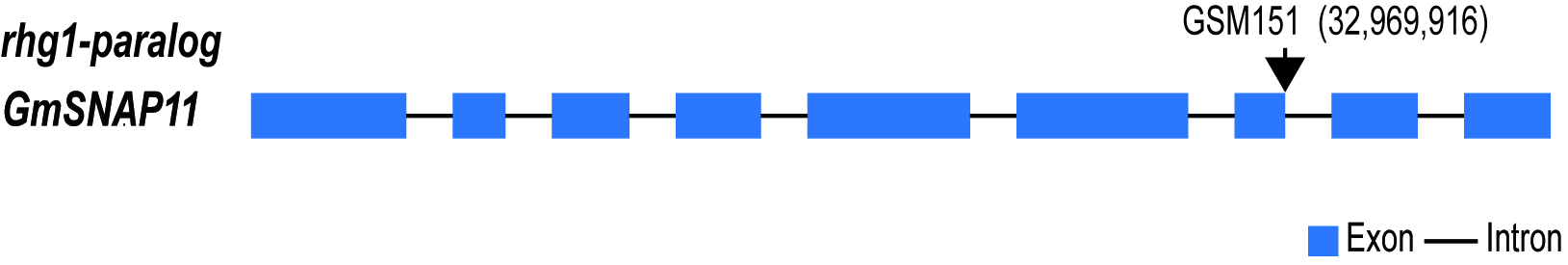


**Figure S1** Position of SNP in *GmSNAP11* at *rhg1-paralog* locus. GSM number was used in the designation of the SNP marker. Blue rectangles and black lines represent exons and introns respectively. The genome location of this SNP is shown (Database version: *Glycine max* Wm82.a2.v1).


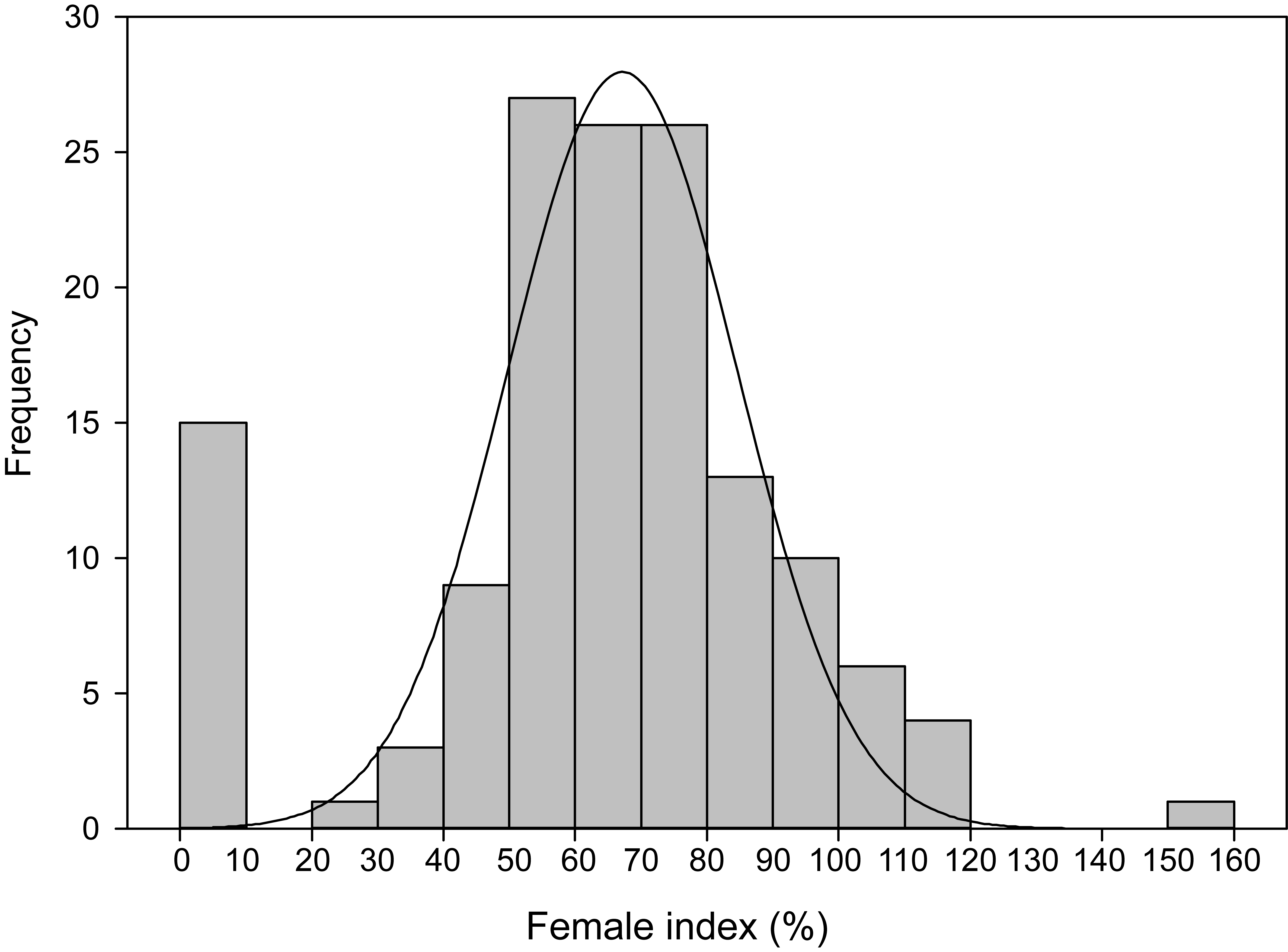


(A)

(B)

**Figure S2** The female index of the RIL population. (A) frequency distribution and (B) phenotypic variability of female index values among the RILs selected for the development of extreme bulks for SCN. A significant difference was observed at *P* < 0.0001. P2 represents the resistant parent (HPD) and P1 represents the susceptible parent (JD23).


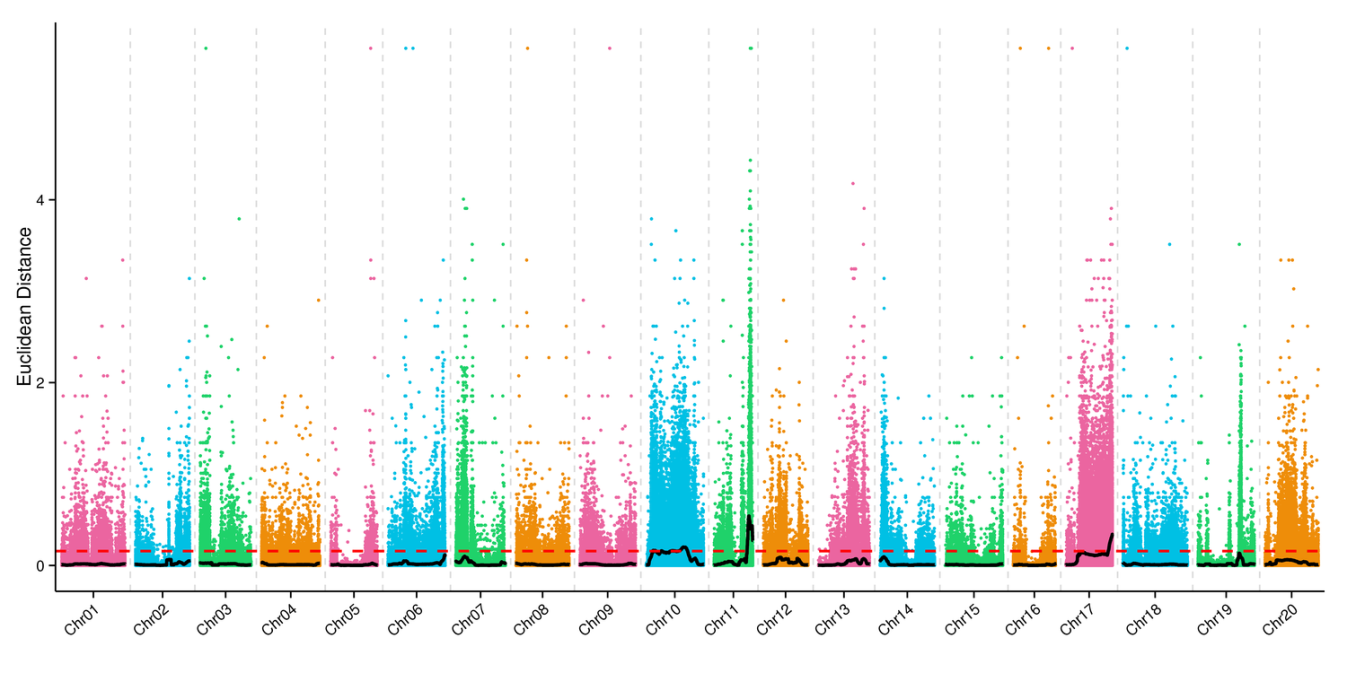


**Figure S3** Euclidean distance analysis. The x-axis represents the positions on the 20 soybean chromosomes and the y-axis represents the Euclidean distance. The black curve shows the Loess fitted curve.


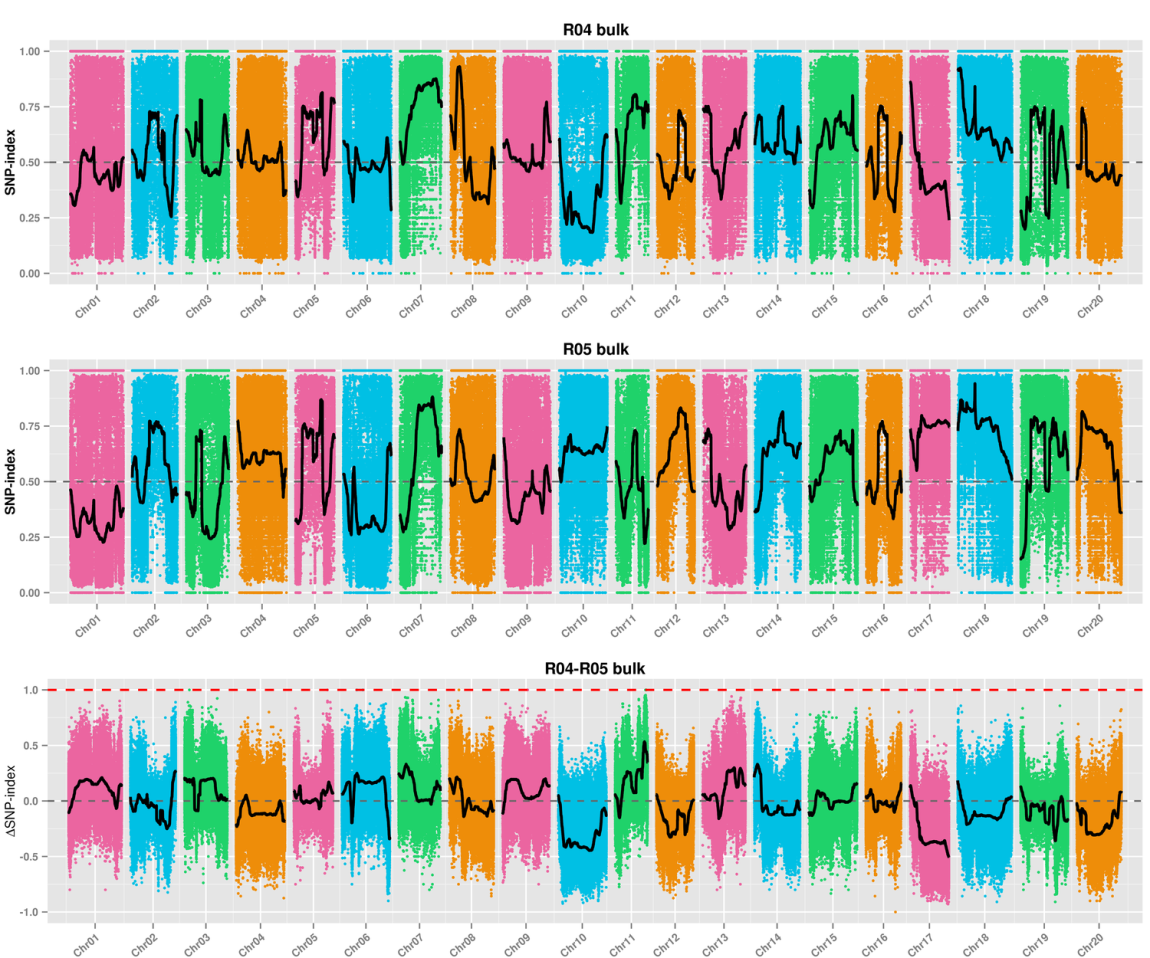


(C)

(B)

(A)

**Figure S4** SNP-index graphs of the R-bulk (**A**), S-bulk (**B**), and Δ (SNP index) (**C**). The x-axis represents the positions on the 20 soybean chromosomes and the y-axis represents the SNP-index. R04, Resistant bulk; R05, Susceptible bulk. The black curves represent the fitted curves calculated through the sliding window method.


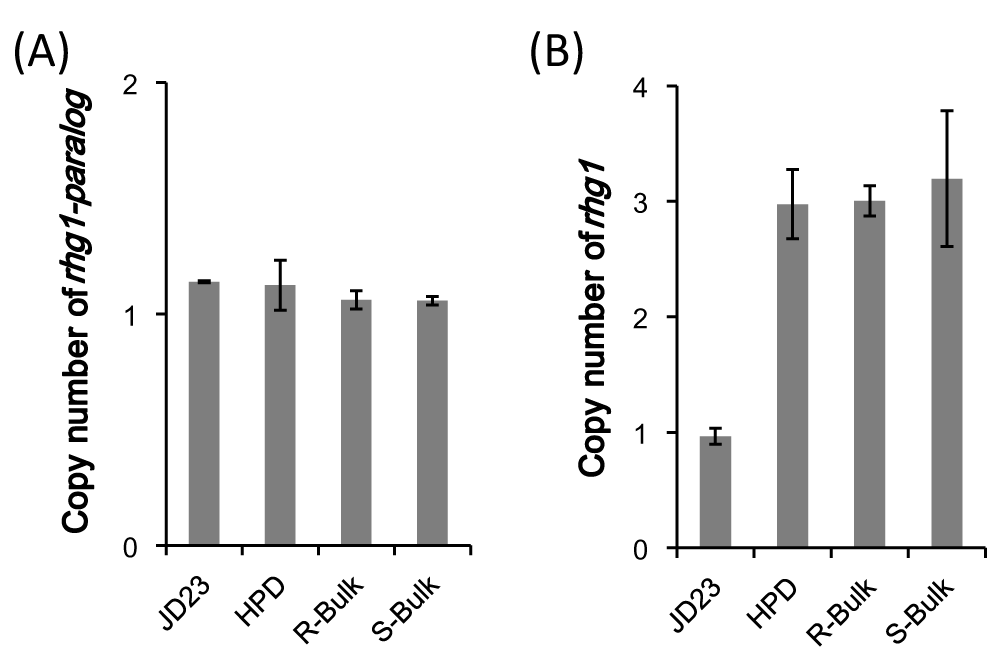


**Figure S5** Copy number variation in various lines. (A) Copy number of *rhg1-paralog* and (B) *rhg1* in JD23, HPD, R-bulk, and S-bulk.

**Figure S6** cDNA sequence alignment of the *GmPLAC8* (*Glyma.11g234400*) gene in parental lines JD23 and HPD showing four SNPs and a deletion producing a frameshift.

**Figure S7** cDNA sequence alignment of the *Glyma.11g234500* (*GmSNAP11*) gene in parental lines JD23 and HPD showing three SNPs and one deletion producing a frameshift mutation.

**Figure S8** Amino acid sequence alignment of *GmSNAP11* in parental lines JD23 and HPD.

**Figure S9** cDNA sequence alignment of *GmSNAP11* in the parental line HPD and four PI lines.


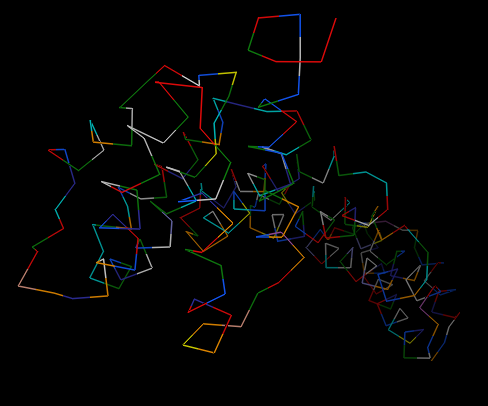

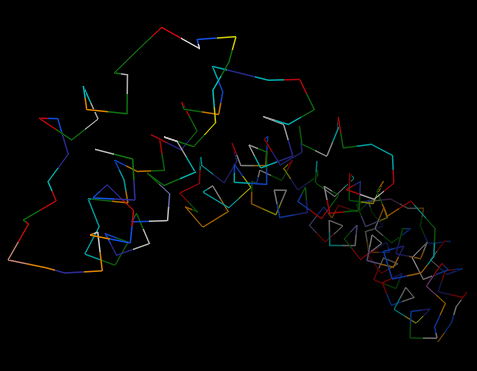


(B)

(A)

**Figure S10** Predicted 3D protein structure of GmSNAP11. Protein structure in (A) JD23 and (B) HPD.


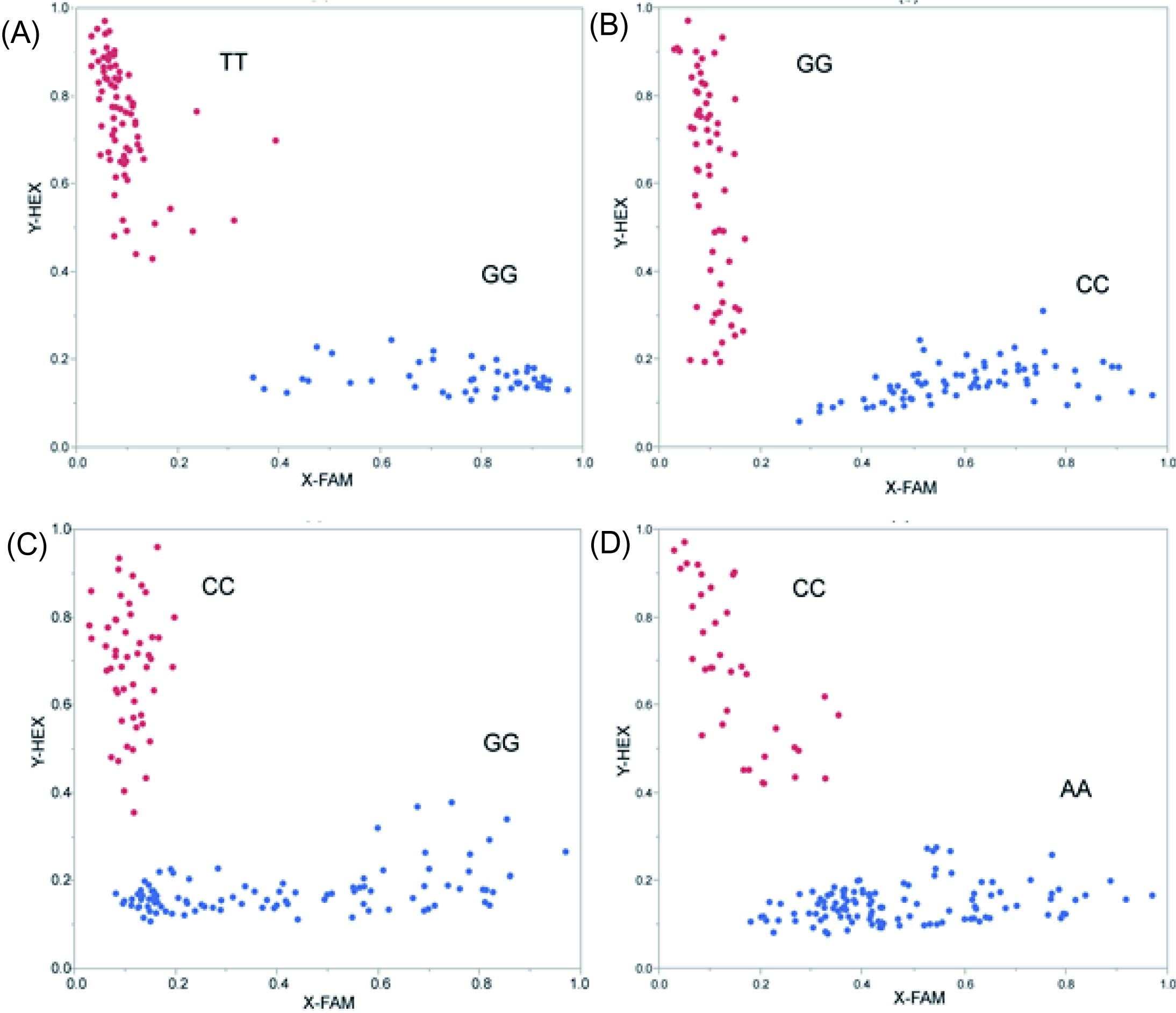


**Figure S11** Endpoint fluorescence scatter plots of the KASP assays. Analysis of (A) GSM381 for *rhg1*; [GG = Peking/PI 88788: TT = Williams 82] (B) GSM383 for *rhg1*; [GG = Peking: CC = Williams 82/PI 88788] (C) GSM150 for *Rhg4*; [CC = Peking: GG = Williams 82] and (D) GSM151 for *rhg1*-*paralog* [AA = Peking: CC = Williams 82] in a set of 145 RILs with known reactions to SCN. Allele-specific primer 1 was labelled with FAM (blue) and allele-specific primer 2 was labelled with HEX (red).
