# Supplementary material for: The GmSNAP11 Contributes to Resistance to Soybean Cyst Nematode Race 4 in Glycine max
Source: Front Plant Sci. 2022 Jul 4;13:939763. doi: 10.3389/fpls.2022.939763 (PMC9289622; doi:10.3389/fpls.2022.939763)
Supplement: Supplementary file 2 [file Data_Sheet_2.docx]

**Table S1** Primers used for gene cloning, vector construction, and qPCR

| Gene | Primer sequence |
| --- | --- |
| **Cloning** |  |
| *Glyma.11g234400* F | ACCATTGATGATACTACGGTTCTG |
| *Glyma.11g234400* R | ACATTATTTGCTAAAGGCAAGATG |
| *Glyma.11g234500* F | GCATTCGATCAATCCATCCATCT |
| *Glyma.11g234500* R | AGTGTAAATCATCAAACAATAGGTCC |
| *Glyma.18G022500* F | CGCTTATGAATCTTCTTCTTCTTC |
| *Glyma.18G022500* R | CCAATTCAATTAAAACCAAAGCAGG |
| **Overexpression (OE)** |  |
| *Glyma.11g234400* F | TGACCATGGTAGATCTGGTTTCGGTTGATAATGGGATTGG |
| *Glyma.11g234400* R | ATTCGAGCTGGTCACCTCATTCTCTTTGTATCAATGAAGG |
| *Glyma.11g234500* F | TGACCATGGTAGATCTGGCCGATCAGTTATCCAAAGGAGAG |
| *Glyma.11g234500* R | ATTCGAGCTGGTCACCTTAAGTAAGATCATCCTCCTCAAG |
| *Glyma.18G022500* F | TGACCATGGTAGATCTGGCCGATCAGTTATCGAAGGGAG |
| *Glyma.18G022500* R | ATTCGAGCTGGTCACCTCAAGTAAGATCATCCTCCTCA |
| **RNAi** |  |
| *Glyma.11g234400* RNAi-forward F | TGACCATGGTAGATCTCTACCACCTTGTTCTTCTGATCAG |
| *Glyma.11g234400* RNAi-forward R | ATTAAGCTGGGACTAGTGGTGTACAAACCAGCAATTGCAGG |
| *Glyma.11g234400* RNAi-Reverse F | AATTCGAGCTGGTCACCCTACCACCTTGTTCTTCTGATCAG |
| *Glyma.11g234400* RNAi-Reverse R | TGCTTCTGCGACGCGTGGTGTACAAACCAGCAATTGCAGG |
| *Glyma.11g234500* RNAi-forward F | TGACCATGGTAGATCTATGGCCGATCAGTTATCCAAAG |
| *Glyma.11g234500* RNAi-forward R | ATTAAGCTGGGACTAGTTTATATTAGTTTTTTTATAGCT |
| *Glyma.11g234500* RNAi-Reverse F | AATTCGAGCTGGTCACCATGGCCGATCAGTTATCCAAAG |
| *Glyma.11g234500* RNAi-Reverse R | TGCTTCTGCGACGCGTTTATATTAGTTTTTTTATAGCT |
| *Glyma.18G022500* RNAi-forward F | TGACCATGGTAGATCTTACGAGGGTGAACAGAATATTGAG |
| *Glyma.18G022500* RNAi-forward R | ATTAAGCTGGGACTAGTCCAGTTCCTGATATCGTTCTAATG |
| *Glyma.18G022500* RNAi-Reverse F | AATTCGAGCTGGTCACCTACGAGGGTGAACAGAATATTGAG |
| *Glyma.18G022500* RNAi-Reverse R | TGCTTCTGCGACGCGTCCAGTTCCTGATATCGTTCTAATG |
| **OE sequencing** |  |
| pGFPGUS-OE-seq F | GACGATTTAGAGTGTTTTACCAAGA |
| pGFPGUS-OE-seq R | TCCCGCCTTCAGTTTAGCTTCATGG |
| **RNAi sequencing** |  |
| I-F2 | CAGCTTAATATGACTCTCAA |
| I-R2 | CAGAAGCAACCTCATGGAAA |
| **qPCR RNAi** |  |
| *Glyma.11g234400* F | TGTTGAGTTTTACAATTTTAATGTG |
| *Glyma.11g234400* R | GTATAATCATTCACTGATGCCTATC |
| *Glyma.11g234500* F | ACGCATTTCTGAAAACCCCTTAC |
| *Glyma.11g234500* R | AACAACAAGAAGAGGATTCGTAAAC |
| *Glyma.18G022500* F | AAAATCGGAAATGGACAATC |
| *Glyma.18G022500* R | TTCATAAGCGAAGGAGCAAA |
| **qPCR OE** |  |
| *Glyma.11g234400* F | CTAAGTGGAGTGGAGGAATA |
| *Glyma.11g234400* R | GAAGTCATAGGCAGGTAAAT |
| *Glyma.11g234500* F | AGGCTCTTGTTTACTATGTG |
| *Glyma.11g234500* R | TTATTGTTGAGGGATTGGCG |
| *Glyma.18G022500* F | CGCCAATCCCTCAACAATAA |
| *Glyma.18G022500* R | GCAATGTCCGCCAACAATCT |
| *GmActin*-F | CGGTGGTTCTATCTTGGCATC |
| *GmActin*-R | GTCTTTCGCTTCAATAACCCTA |

**Table S2** Primers used for the KASP assay of SCN resistance loci *rhg1*, *Rhg4*, and *rhg1-paralog*

| Marker | Sequence |
| --- | --- |
| *Rhg4* GSM150 FAM: | GAAGGTGACCAAGTTCATGCTGAGGTGGCCGCCGGAGG |
| *Rhg4* GSM150 HEX | GAAGGTCGGAGTCAACGGATTGAGGTGGCCGCCGGAGC |
| *Rhg4* GSM150 Common R | CAATCCTGGGGCGTCAACGTC |
| *rhg1-paralog* GSM151 HEX | GAAGGTCGGAGTCAACGGATTCGAAATACCAAAACTAGTGACCTAC |
| *rhg1-paralog* GSM151 FAM | GAAGGTGACCAAGTTCATGCTCGAAATACCAAAACTAGTGACCTAA |
| *rhg1-paralog* GSM 151 Common R | ATCAGGTCTAAGTTTTTTCAATAGTCA |
| *rhg1* GSM381 FAM | GAAGGTGACCAAGTTCATGCTAGCCAAAGAACTTGAGSAGBATGAG |
| *rhg1* GSM381 HEX | GAAGGTCGGAGTCAACGGATTAGCCAAAGAACTTGAGSAGBATGAT |
| *rhg1* GSM381 Common R | CAAACAATAGGTCCAACCACCA |
| *rhg1* GSM383 FAM | GAAGGTGACCAAGTTCATGCTATCTGCMAACTCTGTAAAGAGGAC |
| *rhg1* GSM383 HEX | GAAGGTCGGAGTCAACGGATTATCTGCMAACTCTGTAAAGAGGAG |
| *rhg1* GSM383 Common R | GCTGTCCAGTCTCCAGAAGTGAA |

**Table S3** Reactions of RILs to SCN based on pyramiding of the *rhg1*, *Rhg4*, and *rhg1-paralog* loci (+ = present; - = absent)

| RIL ID | *rhg1* | *Rhg4* | *rhg1-paralog* | FI % |
| --- | --- | --- | --- | --- |
|  |  |  |  |  |
| L1 (JD23) | - | - | - | 78.50 |
| L2 (HPD) | + | + | + | 1.20 |
| L117 | + | + | + | 1.13 |
| L96 | + | + | + | 1.27 |
| L143 | + | + | + | 1.33 |
| L141 | + | + | + | 1.47 |
| L109 | + | + | + | 1.60 |
| L111 | + | + | + | 2.00 |
| L136 | + | + | + | 2.27 |
| L62 | + | + | + | 3.03 |
| L85 | + | + | + | 3.17 |
| L110 | + | + | + | 3.47 |
| L137 | + | + | + | 4.33 |
| L9 | + | + | + | 5.37 |
| L46 | + | + | + | 7.53 |
| L139 | + | + | + | 28.27 |
| L5 | + | + | + | 37.57 |
| L60 | + | + | + | 50.27 |
| L59 | + | + | + | 53.67 |
| L17 | + | + | + | 53.83 |
| L4 | + | + | + | 53.83 |
| L58 | + | + | + | 54.70 |
| L23 | + | + | + | 65.77 |
| L12 | + | + | + | 77.20 |
| L19 | + | + | + | 81.67 |
| L145 | + | + | + | 94.93 |
| L65 | + | + | + | 105.73 |
| L106 | + | + | - | 48.57 |
| L79 | + | + | - | 58.00 |
| L116 | + | + | - | 59.43 |
| L130 | + | + | - | 60.60 |
| L97 | + | + | - | 62.83 |
| L99 | + | + | - | 66.52 |
| L45 | + | + | - | 68.18 |
| L29 | + | + | - | 85.33 |
| L128 | + | + | - | 86.63 |
| L42 | + | - | + | 44.49 |
| L71 | + | - | + | 51.37 |
| L20 | + | - | + | 54.50 |
| L57 | + | - | + | 54.87 |
| L34 | + | - | + | 57.20 |
| L64 | + | - | + | 57.67 |
| L78 | + | - | + | 57.93 |
| L15 | + | - | + | 58.10 |
| L81 | + | - | + | 59.40 |
| L101 | + | - | + | 61.02 |
| L33 | + | - | + | 65.22 |
| L61 | + | - | + | 65.97 |
| L56 | + | - | + | 67.33 |
| L6 | + | - | + | 69.57 |
| L86 | + | - | + | 69.60 |
| L36 | + | - | + | 72.03 |
| L63 | + | - | + | 72.80 |
| L84 | + | - | + | 73.57 |
| L102 | + | - | + | 75.57 |
| L115 | + | - | + | 77.23 |
| L98 | + | - | + | 84.93 |
| L31 | + | - | + | 85.87 |
| L40 | + | - | + | 88.50 |
| L138 | + | - | + | 89.70 |
| L10 | + | - | + | 92.77 |
| L14 | + | - | + | 104.70 |
| L18 | + | - | - | 104.23 |
| L105 | - | + | + | 34.57 |
| L53 | - | + | + | 40.72 |
| L52 | - | + | + | 41.02 |
| L48 | - | + | + | 47.12 |
| L49 | - | + | + | 51.60 |
| L3 | - | + | + | 53.63 |
| L90 | - | + | + | 56.00 |
| L88 | - | + | + | 57.53 |
| L83 | - | + | + | 59.60 |
| L77 | - | + | + | 60.87 |
| L113 | - | + | + | 69.54 |
| L134 | - | + | + | 71.60 |
| L122 | - | + | + | 72.50 |
| L30 | - | + | + | 74.53 |
| L91 | - | + | + | 90.43 |
| L129 | - | + | + | 92.20 |
| L92 | - | + | + | 96.40 |
| L120 | - | + | + | 96.67 |
| L32 | - | + | + | 105.77 |
| L144 | - | + | + | 114.20 |
| L70 | - | + | - | 53.37 |
| L73 | - | + | - | 60.80 |
| L127 | - | + | - | 71.20 |
| L119 | - | + | - | 71.83 |
| L100 | - | + | - | 77.83 |
| L27 | - | + | - | 79.47 |
| L68 | - | + | - | 97.60 |
| L41 | - | - | + | 34.62 |
| L8 | - | - | + | 42.27 |
| L50 | - | - | + | 43.08 |
| L80 | - | - | + | 44.67 |
| L43 | - | - | + | 48.38 |
| L93 | - | - | + | 48.47 |
| L54 | - | - | + | 51.43 |
| L39 | - | - | + | 56.07 |
| L123 | - | - | + | 58.03 |
| L76 | - | - | + | 60.07 |
| L51 | - | - | + | 60.79 |
| L28 | - | - | + | 61.50 |
| L89 | - | - | + | 62.27 |
| L126 | - | - | + | 65.40 |
| L37 | - | - | + | 66.59 |
| L67 | - | - | + | 68.10 |
| L44 | - | - | + | 68.32 |
| L22 | - | - | + | 68.77 |
| L26 | - | - | + | 69.07 |
| L38 | - | - | + | 69.17 |
| L11 | - | - | + | 69.43 |
| L142 | - | - | + | 72.33 |
| L7 | - | - | + | 73.73 |
| L133 | - | - | + | 74.80 |
| L47 | - | - | + | 75.93 |
| L125 | - | - | + | 76.40 |
| L95 | - | - | + | 77.33 |
| L72 | - | - | + | 78.57 |
| L69 | - | - | + | 79.10 |
| L75 | - | - | + | 80.47 |
| L103 | - | - | + | 82.19 |
| L112 | - | - | + | 83.37 |
| L35 | - | - | + | 83.60 |
| L13 | - | - | + | 85.43 |
| L124 | - | - | + | 90.10 |
| L24 | - | - | + | 94.20 |
| L55 | - | - | + | 95.63 |
| L21 | - | - | + | 100.37 |
| L25 | - | - | + | 104.77 |
| L121 | - | - | + | 109.50 |
| L131 | - | - | + | 111.77 |
| L132 | - | - | + | 113.27 |
| L16 | - | - | + | 118.23 |
| L108 | - | - | - | 58.60 |
| L94 | - | - | - | 58.83 |
| L74 | - | - | - | 59.20 |
| L135 | - | - | - | 70.13 |
| L87 | - | - | - | 71.17 |
| L118 | - | - | - | 71.57 |
| L114 | - | - | - | 72.35 |
| L107 | - | - | - | 80.06 |
| L66 | - | - | - | 85.97 |
| L104 | - | - | - | 87.54 |
| L82 | - | - | - | 156.77 |

**Table S4** Information about the clean reads obtained by whole-genome resequencing

| ID | Clean reads | Clean bases | Q30 (%) | GC (%) |  |
| --- | --- | --- | --- | --- | --- |
| R01 | 145,488,663 | 43,909,303,958 | 88.44 | 36.85 |  |
| R04 | 125,674,889 | 31,652,235,434 | 94.82 | 37.73 |  |
| R05 | 146,680,637 | 36,943,134,474 | 95.00 | 37.14 |  |
| R06 | 186,063,811 | 46,869,047,456 | 95.19 | 37.33 |  |

R01: JD23; R04: Resistant bulk; R05: Susceptible bulk; R06: HPD

**Table S5** Summary of sequencing data

| Sample | Average depth | Cov_ratio_1× (%) | Cov_ratio_5× (%) | Cov_ratio_1× (%) |
| --- | --- | --- | --- | --- |
| JD23 | 40 | 98.33 | 97.37 | 96.32 |
| R-bulk | 27 | 99.16 | 98.17 | 95.53 |
| S-bulk | 32 | 99.16 | 98.37 | 96.89 |
| HPD | 41 | 98.06 | 96.65 | 95.17 |

**Table S6** Summary of chromosome regions identified by Euclidean distance (ED) analysis

| Chromosome ID | Start | End | Size (Mb) | Gene Number |
| --- | --- | --- | --- | --- |
| Chr10 | 13,350,000 | 14,180,000 | 0.830 | 28 |
| Chr10 | 22,380,000 | 22,580,000 | 0.200 | 1 |
| Chr10 | 22,630,000 | 22,640,000 | 0.010 | 2 |
| Chr10 | 22,660,000 | 26,760,000 | 4.10 | 101 |
| Chr10 | 29,150,000 | 37,250,000 | 8.10 | 321 |
| Chr10 | 5,980,000 | 6,780,000 | 0.800 | 81 |
| Chr10 | 7,470,000 | 8,860,000 | 1.39 | 70 |
| Chr11 | 29,680,000 | 34,760,000 | 5.08 | 796 |
| Chr17 | 37,930,000 | 41,640,000 | 3.71 | 608 |
| Total | - | - | - | 2,008 |

**Table S7** Summary of chromosome regions identified by ∆SNP-index analysis

| Chromosome ID | Start | End | Size (Mb) | Gene Number |
| --- | --- | --- | --- | --- |
| Chr07 | 6,890,000 | 7,470,000 | 0.580 | 77 |
| Chr07 | 7,570,000 | 9,200,000 | 1.63 | 244 |
| Chr11 | 29,500,000 | 34,760,000 | 5.26 | 799 |
| Chr14 | 2,480,000 | 4,400,000 | 1.92 | 325 |
| Chr14 | 4,460,000 | 4,460,000 | 0.00 | 2 |
| Chr14 | 4,500,000 | 4,510,000 | 0.010 | 1 |
| Chr14 | 4,550,000 | 4,570,000 | 0.020 | 4 |
| Total | - | - | - | 1,452 |

**Table S8** Overlapping genomic regions associated with SCN4 resistance based on ED and ∆SNP-index results

| Chromosome ID | Start | End | Size (Mb) |
| --- | --- | --- | --- |
| Chr11 (ED) | 29,680,000 | 34,760,000 | 5.08 |
| Chr11 (∆SNP-index) | 29,500,000 | 34,760,000 | 5.26 |
| Chr11 (Overlap) | 29,680,000 | 34,760,000 | 5.08 |

**Table S9** Numbers of SNPs observed between the two parents

| Type | Number of SNPs |
| --- | --- |
| UTR | 241 |
| Upstream | 3,009 |
| Synonymous | 214 |
| Splice site | 31 |
| Start codon | 26 |
| Stop codon | 2 |
| Nonsynonymous | 237 |
| Intron | 1493 |
| Intergenic | 1368 |
| Downstream | 1807 |
| Total | 8,428 |

**Table S10** Reactions of 83 soybean accessions harbouring *rhg1*, *Rhg4*, and *rhg1-paralog* to SCN4

| **Accession** | **Reaction** | **Accession** | **Reaction** | **Accession** | **Reaction** | **Accession** | **Reaction** |
| --- | --- | --- | --- | --- | --- | --- | --- |
| WDD03084 | R | WDD01613 | R | ZDD10057 | R | Qihuang38 | S |
| WDD03003 | R | WDD03052 | R | ZDD10060 | R | Qihuang40 | S |
| ZDD01060 | R | WDD01659 | R | ZDD07948 | R | ZDD01855 | S |
| ZDD01412 | R | WDD00640 | R | Zhonghuang57 | R | ZDD01884 | S |
| ZDD02255 | R | WDD03008 | R | ZDD10251 | R | ZDD01890 | S |
| ZDD02258 | R | WDD01614 | R | ZDD02226 | R | ZDD01898 | S |
| ZDD02370 | R | ZDD02252 | R | ZDD02315 | R | ZDD01909 | S |
| ZDD02450 | R | ZDD08257 | R | ZDD02255 | R | ZDD02967 | S |
| ZDD08251 | R | ZDD01818 | R | PI437654 | R | ZDD08460 | S |
| ZDD10254 | R | ZDD01857 | R | WDD02019 | S | ZDD08489 | S |
| ZDD01861 | R | ZDD01858 | R | WDD03116 | S | ZDD08493 | S |
| ZDD03683 | R | ZDD01892 | R | WDD00661 | S | ZDD10076 | S |
| ZDD08494 | R | ZDD01922 | R | WDD01645 | S | ZDD10801 | S |
| ZDD08537 | R | ZDD03056 | R | WDD01655 | S | ZDD11436 | S |
| ZDD19379 | R | ZDD08483 | R | WDD00467 | S | ZDD11461 | S |
| PI43848913 | R | ZDD08487 | R | ZDD01399 | S | ZDD18394 | S |
| WDD02998 | R | ZDD08488 | R | ZDD18018 | S | ZDD08472 | S |
| WDD02989 | R | ZDD08502 | R | ZDD23221 | S | PI84751 | S |
| WDD01583 | R | ZDD08505 | R | ZDD08250 | S | Manokin | S |
| WDD01623 | R | ZDD08510 | R | ZDD10293 | S | PI90763 | S |
| WDD01622 | R | ZDD08511 | R | ZDD18512 | S |  |  |
